# ENGAGING IN ARTISTIC AND CREATIVE OPPORTUNITIES TO HELP UNDERSTAND THE AGING EXPERIENCE

**DOI:** 10.1093/geroni/igad104.0786

**Published:** 2023-12-21

**Authors:** Alexander Bishop, Melinda Heinz, Victor Fung

## Abstract

This symposium provides an overview of artistic and creative engagement methodologies to understand aging experiences. The first presentation examines a collaborative effort involving a researcher and filmmaker to film, edit, and produce a documentary about Irish Men’s Sheds to increase awareness of diversity within the dissemination of narrative stories. The second presentation focuses on drawing, investigating how young children socially and cognitively represent perceptions of human longevity and of the concept of what constitutes “old.” Children were asked questions regarding what it means to be “old” and what they might look like at 100 years, and to draw a self-portrait image. Findings demonstrating internal working models consistent with socially-learned attitudes, beliefs, and stereotypes of aging. The third presentation explains digital narrative gerontology and a life story project of the older adult’s life created by younger and older adults. Thematic analysis revealed improved well-being, reduced generational stereotypes, and feelings of loneliness and isolation. The fourth presentation details an intergenerational project involving university students and older adults with cognitive impairment. Participants engaged in a six-week, telephone-based reminiscence experience and then co-created a storyboard/script to create a digital story. Thematic analysis demonstrated six themes: Family, religion and purpose in life, loves and hates, career/work, stress/coping, and major life turning points. The final presentation details music as a therapeutic intervention to improve older adults’ quality of life and foster social connections, self-understanding and expression. Observations from working with older adults in a variety of environments and preferred music styles will also be discussed.

